# Staff Training to Improve the Preschool Educational Environment for Children with Special Needs

**DOI:** 10.1007/s40617-025-01123-3

**Published:** 2025-12-19

**Authors:** Hege Tryggestad, Sigmund Eldevik, Svein Eikeseth, Børge Strømgren, Sheri Kingsdorf

**Affiliations:** 1 Departement of Behavioral Science, Oslo Metropolitan University, Oslo, Norway; 2 Institute for Research in Inclusive Education, Masaryk University, Brno, Czech Republic

**Keywords:** Staff training, EIBI, APERS-P-SE, Quality of the teaching and learning environment, Special education

## Abstract

Ensuring high-quality general and special education in preschools is crucial for the positive development of at-risk children and children with special needs. However, recent reports suggest inconsistencies in general education quality and challenges in implementing special education support and early intensive behavioral intervention (EIBI). These results indicate that further training is needed to improve the quality of services for preschool children with special needs. The current study employed a delayed multiple probe design to evaluate a staff training program’s influence on the quality of general education, EIBI, and evocative situations in three inclusive preschool units. The intervention lasted 17 weeks, consisting of a 4-h post-baseline workshop and biweekly coaching with the entire staff group. The quality of the teaching and learning environment, measured by the Autism Program Environment Rating Scale (APERS-P-SE), improved in two preschools. We observed improvement in the quality of evocative situations with a large effect size, increased children’s response rate during lunch, improved EIBI quality, and good social validity. This indicates that using APERS-P-SE to establish objectives for inclusive practices and training the entire staff group with a focus on evocative situations may be one way to improve the implementation of EIBI and the quality of preschools’ teaching and learning environment.

The increasing number of children with autism spectrum disorder (ASD) and intellectual disabilities in mainstream early childhood education settings presents significant challenges for preschool staff and caregivers (Bejnö et al., 2019). Effective inclusive practices require the systematic integration of general and special education service delivery models, achieved through structured collaboration among educators and support staff (Odom et al., 2011). This coordination ensures consistency in instructional strategies and intervention implementation across settings, individuals, and activities (Odom et al., 2011). A key component of inclusive education is the implementation of evidence-based practices (EBPs); however, many early childhood educators report limited training and support in this area (Layden et al., 2024). Despite differences in education models around the world, this dearth of training and support has been observed in numerous countries (e.g., Norway, the US, and Sweden), as noted by several studies (Eldevik et al., 2019; Layden et al., 2024; Roll-Pettersson et al., 2016).

In Norway, where the present study was conducted, 94.4% of children aged 1–5 attend state-subsidized preschools, with near-universal enrollment among 3–5-year-olds (Statistics Norway, 2025). Staffing regulations specify general and pedagogical staffing ratios (Regulation on Pedagogical Staffing & Exemptions in Preschools, 2017 §1). Children requiring additional support have the right to special education, determined through expert assessment by the local Pedagogical-Psychological Services (PPT). For children with ASD, they may recommend evidence-based interventions such as Early Intensive Behavioral Intervention (EIBI).

EIBI is a well-established intervention for children with ASD (Reichow et al., 2018; Rodgers et al., 2021), with evidence suggesting long-term benefits (Perry et al., 2019; Smith et al., 2019). EIBI targets socially significant behaviors across multiple domains, including communication, daily living skills, and social interactions (Bejnö et al., 2021; Klintwall & Eikeseth, 2014). However, successful implementation of community-based EIBI faces multiple barriers, such as: (a) low levels of acceptance among preschool staff for implementing EIBI, (b) limited knowledge and training in evidence-based approaches, (c) lack of competency and understanding of ASD and EBP, (d) lack of collaboration among special educators, classroom staff, management, and parents, and (e) difficulties in providing the recommended intensity (hours per week) (Eldevik et al., 2019; Långh et al., 2017; Roll-Pettersson et al., 2016; Sinai-Gavrilov et al., 2019).

To address these barriers, it is essential to apply principles from implementation science, which examines methods for integrating evidence-based practices into real-world settings, and to focus on key competency drivers such as staff training and coaching that ensure individuals and organizations have the necessary skills, knowledge, and support for successful implementation (Fixsen et al., 2009; Paquet et al., 2023). In this context, professional development programs aimed at enhancing staff competence have gained increasing attention, with various training formats, such as workshops, video modeling, lectures, and seminars, being widely implemented (Carroll et al., 2022; Schachter, 2015; Reed & Henley, 2015). While a single lecture, workshop, or training session may increase knowledge, it is rarely sufficient to enhance skills or maintain long-term intervention fidelity (Schachter et al., 2019).

Research suggests that ongoing support is necessary to facilitate skill acquisition, promote generalization, and sustain intervention fidelity over time (Fixsen et al., 2009; Novak et al., 2019). Goal setting and coaching with performance feedback may enhance the maintenance and refinement of staff competencies, ultimately improving learning outcomes for both staff and children (McLucas et al., 2024; Novak et al., 2019; Paquet et al., 2023). Among these, performance feedback plays a crucial role in staff training by correcting integrity errors and ensuring high implementation fidelity (Novak et al., 2019; Parsons & Reid, 1995; Reed and Henley. 2015).

To further support staff performance, goal setting functions as an antecedent intervention that establishes specific, measurable objectives through verbal rules that define the relationship between the target goal, required behaviors, and their consequences (Malott, 1992). Its effectiveness depends on clearly defined contingencies in which reinforcement is contingent on goal attainment, with performance feedback often serving as the primary consequence (McLucas et al., 2024). However, isolating the effects of goal setting is challenging, as it is often implemented within multicomponent intervention packages, making it difficult to analyze individual components and their impact on behavior change (McLucas et al., 2024).

Given these complexities, goal setting is often complemented by coaching and supervision to maximize its effectiveness. Coaching provides ongoing feedback and guidance, ensuring that practitioners not only meet performance criteria but also sustain and generalize their skills across contexts (Novak et al., 2019). Coaching and supervision are structured relationship-based processes led by experts to enhance practitioners’ professional skills, behaviors, and competencies (Kucharczyk et al., 2012). These methods support skill acquisition, promote implementation fidelity, and facilitate the generalization of learned strategies to natural contexts through ongoing feedback and guidance (Joyce & Showers, 2002; Snyder et al., 2015). By emphasizing goal setting and achievement, coaching can be adapted for individuals or groups, ensuring targeted and effective professional development (Schachter, 2015). Research has identified coaching and supervision as essential components for the successful implementation of EIBI and for enhancing inclusive practices for children with ASD (Bejnö et al., 2021; Eikeseth et al., 2009; Paquet et al., 2023; Snyder et al., 2018).

Building on the importance of coaching and supervision, behavioral skills training (BST) has emerged as a well-established, evidence-based training package, integrating instruction, modeling, rehearsal, and feedback, with each step repeated until participants master the targeted skills (DiGennaro Reed et al., 2018; Parsons et al., 2013; Slane & Lieberman-Betz, 2021). BST has been successfully applied to teach professionals and caregivers behavior analytic principles and interventions, such as mand training (Nigro-Bruzzi & Sturmey, 2010), discrete-trial teaching (Sarokoff & Sturmey, 2004), the picture exchange communication system (Rosales et al., 2009), and Trial-based functional analysis (Standish et al., 2023) as well as in different formats, including one-on-one and group-based training (Courtemanche et al., 2021).

As previously mentioned, several studies have identified multiple barriers to the implementation of EIBI and the maintenance of high program quality for children with ASD and other disabilities in preschool settings. These barriers raise concerns about whether current service levels in Norway are sufficient to yield meaningful outcomes. While some research has examined the effects of reduced intervention intensity (Eldevik et al., 2019), no studies, to our knowledge, have investigated staff training programs designed for entire preschool units to enhance inclusive practices and EIBI in classrooms with children with ASD or intellectual disabilities in Norway. Although this work focuses on Norway, the challenges encountered and strategies employed are likely relevant across various regions and countries, given the persistent gap between research and practice (Eldevik et al., 2019; Layden et al., 2024; Roll-Pettersson et al., 2016).

To address these challenges, this study examined the impact of an in-service training model by implementing a unit-wide training approach in three preschools. The goal was to enhance the quality of EIBI and improve the overall program quality for children with ASD and other developmental disabilities in inclusive early childhood settings. Specifically, we aimed to answer the following research questions: (1) How does theoretical in-service training, including goal setting and biweekly coaching, influence the quality of EIBI and overall program quality in three inclusive preschools, as measured by the Autism Program Environment Rating Scale Sweden (APERS-P-SE) and the Periodic Service Review (PSR)? (2) What is the effect of BST and biweekly coaching on the accurate implementation of evocative situations (i.e., antecedent-behavior-consequence contingencies) during lunch, as measured by Teacher Performance Rate and Accuracy (TPRA)?

## Method

### Recruitment and Inclusion Criteria

A preschool was eligible for inclusion if they met the following criteria: (a) had a child with special needs who received special education services for at least 20 h per week, (b) the child had at least one year remaining of their preschool education, (c) the child had a referral from the local Educational Psychological Service recommending that the child receive EIBI, and (d) the preschool had an EIBI supervisor assigned.

Initially, the first author provided information about the study to the agency overseeing the preschools in the municipality where the study aimed to take place. Upon obtaining permission from this agency, contact with the preschools and the EIBI supervisors was initiated. They were given comprehensive written details about the study along with consent forms. If the preschool leadership, staff, and EIBI supervisors demonstrated their willingness to engage by supporting the consent forms, comprehensive written details regarding the research, including a consent form, would be provided to parents or legal guardians of children with special needs.

### Participants

#### The Participating Preschool Staff

Three preschools met the eligibility criteria and participated in the study. In preschool A, four female employees with an average age of 40 years and a mean of 12 years of experience in the preschool environment worked in the targeted unit (range 4–21 years). All had prior experience working with children with special needs (M = 6 years, range 3–10 years), two held bachelor’s degrees, one functioned as a special educator 20 h a week, and one employee had no education beyond high school.

Preschool B employed six individuals in the targeted unit, five females and one male, with an average age of 53. They collectively possessed extensive experience within the preschool setting, averaging 21 years (range 11–40 years) and an average of 5 years (range 1–12 years) working with children with special needs. Two held reduced positions or temporarily filled in for colleagues on sick leave, three held bachelor’s degrees, two worked as special educators 20 h a week, and one staff member did not have a college degree.

Preschool C employed four individuals in the targeted unit, two females and two males, with an average age of 41. They had an average of 14 years of experience in the preschool setting (range 7–22 years) and 6.5 years of experience (range 2–10 years) working with children with special needs. Two employees held bachelor’s degrees as preschool teachers, one worked as a special educator 20 h a week, and one staff member without a college degree served as an assistant.

Some staff members left or went on maternity leave during the preschool year. In preschool A, one left the preschool during the project, and another went on maternity leave two weeks before the follow-up assessment. In preschool B, two part-time employees left during the project period. They were replaced by two assistants on temporary contracts. In preschool C, all employees continued their work in the preschool during all study phases.

#### The Participating Children

The child participant from preschool A was a 32-month-old girl diagnosed with ASD. She came from a multilingual home where Urdu, English, and Norwegian were spoken. Assessment and observations showed limited expressive and receptive language. Additionally, she exhibited few vocal and nonvocal imitation skills and did not use augmentative and alternative communication (AAC). During free play, she frequently engaged in stereotypic behaviors, such as pounding two bricks together while moving around the room. She also displayed episodes of self-injurious behavior, including head banging toward the floor and other challenging behaviors like intense crying and object throwing. The child’s individualized education plan (IEP) included objectives such as: (a) matching objects, (b) joint attention, (c) mand, (d) listener behavior, (e) imitation, (f) play, and (g) self-help skills. At the study’s outset, she had received EIBI for three months. On the Childhood Autism Rating Scale 2 (CARS2) (Schopler et al., 2010), she received a raw score of 49.5, indicating severe symptoms of autism.

The child participant from preschool B was a 51-month-old boy diagnosed with ASD. He came from a multilingual home where Bilen, Tigrinya, English, and Norwegian were spoken. Assessment results and observations indicated that although he could vocalize words and sentences, he had minimal functional communication skills and did not use AAC. His receptive language was approximately 100 words. He frequently engaged in stereotypic behaviors, including hand flapping and spinning, and frequently moved between activities during free play. His IEP goals included: (a) matching objects and pictures, (b) joint attention, (c) mand, (d) listener behavior, (e) vocal imitation, (f) play, and (g) self-help skills. At the onset of the study, he had undergone ten months of EIBI. Initial assessment on the CARS2 yielded a score of 40.5, indicating severe symptoms of autism.

The child participant in preschool C was a 58-month-old girl experiencing significant developmental delays in language, social interaction, and motor skills. Her cognitive and adaptive skills indicated functioning below the average level. She came from a bilingual home where Berber and Norwegian were spoken. Observations revealed that she had some vocal speech, including words like “mummy” and “daddy,” and could use some hand signs such as “bicycle” and “sing.” However, her functional communication skills were limited, with some reliance on sign language. Her IEP goals focused on: (a) mand, (b) listener behavior, (c) motor imitation, (d) play, and (e) self-help skills. At the beginning of the study, she had received three months of EIBI. Her initial assessment using CARS2 yielded a score of 20, suggesting minimal to no symptoms of autism.

#### Design

The study employed a delayed multiple probe design across participants to evaluate the intervention effects (Slocum et al., 2022). The preschools were randomly assigned by lottery, by two independent bachelor students to baseline lengths of three, four, and five weeks. The various outcome measures were administered during baseline, intervention, post-intervention, and follow-up from October to June.

#### Setting

We conducted the study in three mainstream Norwegian-speaking public preschools in Norway. These preschools were situated within a single municipality with varied socio-economic levels. However, the proportion of children in low-income households was below the country average. Preschools A and B were the largest, with four units accommodating 68 children. Preschool C had two units and served 31 children aged 1 to 6. All the preschools had a large classroom with one or two group rooms. The classrooms contained tables, chairs for activities and meals, and shelves with toys and materials. The daily schedule in the preschools typically included: (a) arrival, (b) breakfast, (c) free play, (d) small group activities, (e) circle time, (f) meal, (g) outdoor play, (h) meal, (i) play, and (j) departure. Lunchtime was designed as a free operant setting where the child and staff could engage in a behavior at any time. Typically, approximately 20–24 children had lunch together in groups of six to eight children per table, with an employee seated alongside the participating child to provide mealtime assistance. In addition, there was one staff member at each table.

Mealtimes are everyday situations and essential routines for new learning and generalizing skills such as mand, listener behavior, independence, and social skills (Klette et al., 2016), behaviors included in the IEPs of all children in the current study.

Lunchtime was chosen as a setting for coaching and assessing evocative situations for several reasons: (a) meals are considered an essential daily activity for the staff; (b) multiple staff members are present simultaneously for support and observational learning; (c) children gather around tables with shared attention on eating and social behavior, (d) meals are served two to three times per day, allowing staff to practice their skills several times a day; (e) meals typically have a certain duration that, under the right conditions, might result in numerous evocative situations; and (f) meals can serve as a setting for learning and generalizing skills included in the children’s IEP (e.g., mand and listener behavior).

#### Training Materials

The materials used to conduct the in-service training workshop at the preschools included: (a) a PowerPoint document, (b) a laptop computer, (c) an LCD projector, (d) copies of the performance monitoring tool, and (e) various scripts and stimuli (e.g., toys, datasheets, and writing utensils) for role-playing.

#### Training the Research Team

Prior to delivering the intervention, an external expert from Sweden trained the first author and an EIBI supervisor in using the APERS-P-SE approximately two months before the onset of the baseline. The calibration process lasted approximately two days. The sessions consisted of a theoretical review, practical implementation in one preschool, and discussions to achieve agreement on the rating scale.

The EIBI supervisors attended a two-hour workshop led by the first author on using TPRA approximately two weeks before the onset of the baseline. The first author introduced TPRA with a PowerPoint presentation. Next, role-plays were conducted where the supervisors alternated between the roles of a trainer and the child, while two scored the TPRA form. The tasks to be practiced included mand and listener skills, such as “give me the car.” Role-plays and assessments were conducted to ensure reliability between the first author and the supervisors prior to baseline. All supervisors achieved 100% agreement with the first author. Throughout the project, the first author continued to answer questions concerning APERS-P-SE and TPRA, provide feedback, and discuss situations that arose in the preschools.

### Independent Variable

The independent variable consisted of a 4-h training workshop, one for each preschool, and eight two-hour biweekly coaching sessions. During the 17-week intervention period, the staff in each preschool received 4 h of in-service training and 16 h of coaching. The workshop was divided into three components: (a) theoretical in-service training, which lasted for 1.5 h; (b) feedback on APERS-P-SE and goal selection to improve the teaching and learning environment, which lasted for 1 h; and (c) Behavioral Skills Training (BST) 1.5 h. A 5 to 10-min break was conducted approximately every 45 min. See detailed descriptions of each component below.

The in-service training workshop took place in each preschool’s classroom during November. It was conducted on planning days for preschools A and B, with staff receiving 4 h consecutively. Preschool C had 4 h distributed over two consecutive days, two hours each day. The three workshops had the same structure and content except for each preschool’s target selection discussions based on APERS-P-SE data. The preschool unit staff attended the workshops, including the special education teacher and the EIBI supervisor. Four staff members participated in preschool A, six in preschool B, and four employees in preschool C. However, two part-time employees in preschool B did not participate in the behavioral skills training (BST) component. However, they participated in biweekly coaching sessions, supporting the work to improve the overall educational quality. Biweekly coaching sessions were conducted in the classroom during the unit’s regularly scheduled staff meeting while the children played outside. All classroom staff, including the special education teachers and sometimes EIBI supervisors, attended the mandatory sessions.

#### Component 1: Theoretical In-service Training Session

The theoretical in-service training was conducted in a classroom setting with a lecture and a PowerPoint presentation by the first author, interspersed with questions, participant reflections, and plenary discussions. The theoretical in-service training session contained 9 topics of: (1) inclusion, (2) program quality, (3) evidence-based practice, (4) evocative situations, (5) how to facilitate the environment to promote children’s initiative, (6) mand, (7) listener skills, (8) prompt and prompt/fading, and (9) differential reinforcement.

#### Component 2: Feedback Based on APERS-P-SE and Goal Selection

The first author presented the pretest findings of APERS-P-SE, including strengths and weaknesses, to the staff group. They conveyed their perspectives, evaluated their practices, and further selected three objectives related to the general quality of the preschool. The objectives aimed to support the development of all children in the unit by implementing structured classroom practices, such as organizing the environment into small play areas, conducting daily circle time, or establishing playgroups three days per week. To facilitate gradual implementation, subgoals were systematically structured: (1) initiating small playgroups with four children at least once per week, (2) expanding sessions to Monday and Wednesday, (3) further increasing to Monday, Wednesday, and Thursday, and (4) ensuring that playgroup content was planned and structured during biweekly staff meetings. In addition, the objectives involved more specific changes in line with the child’s IEP, such as: (a) establishing playgroups with three children three days a week, with objectives drawn from the child’s IEP; (b) using visual aids in transition situations, play, and educational activities; and (c) increasing the number and quality of evocative situations throughout the day.

#### Component 3: Behavioral Skills Training (BST)

The BST session was conducted in the classroom by the first author and one EIBI supervisor, with four staff members attending. It consisted of four steps: (a) instructions, (b) modeling, (c) rehearsal, and (d) feedback (DiGennaro Reed et al., 2018). BST was used to train staff on three procedures: (a) how to perform evocative situations, (b) how to contrive and utilize natural learning opportunities effectively, and (c) how to contrive contingencies that evoke mands for preferred items in children. During the roleplay, staff members alternated between trainer and child. As part of the instruction component of the BST procedure, all employees were given a written description of small, operationalized behavior targets based on the performance monitoring tool (PMT) (Kazemi et al., 2018). Before demonstrating the target skills, the first author explained the tasks and carefully guided the staff through the PMT and the roleplay activity. The first author and the EIBI supervisor demonstrated the target skills. Further, the employees conducted roleplay based on the written description and received feedback until they met the mastery criteria of 100% correct responses on six trials (opportunities). The first author and the EIBI supervisor used the TPRA as a basis for providing accurate feedback.

#### Biweekly Coaching

The first biweekly coaching session was conducted two weeks after the initial 4-h workshop. The agenda was to review the staff’s evaluation of the recent period’s work, discuss the preliminary evocative situations data, hands-on supervision, provide feedback, and adjust the objectives and action plans for the next two weeks. The coaching sessions were divided into two parts. In the first part, the first author provided hands-on supervision to the staff member responsible for supervising the child during the meal. The primary focus was on the evocative situations and the procedure’s treatment fidelity. The first author used the TPRA to provide specific feedback. The second part of the coaching sessions was a staff meeting, based on Kucharczyk et al. (2012), following the observation and hands-on supervision during the meal. The aim was to give the entire staff group feedback and direction on implementing evocative situations, as well as to assess progress on the action plan and objectives concerning the quality of general education.

### Dependent Variable

**Fig. 1:**
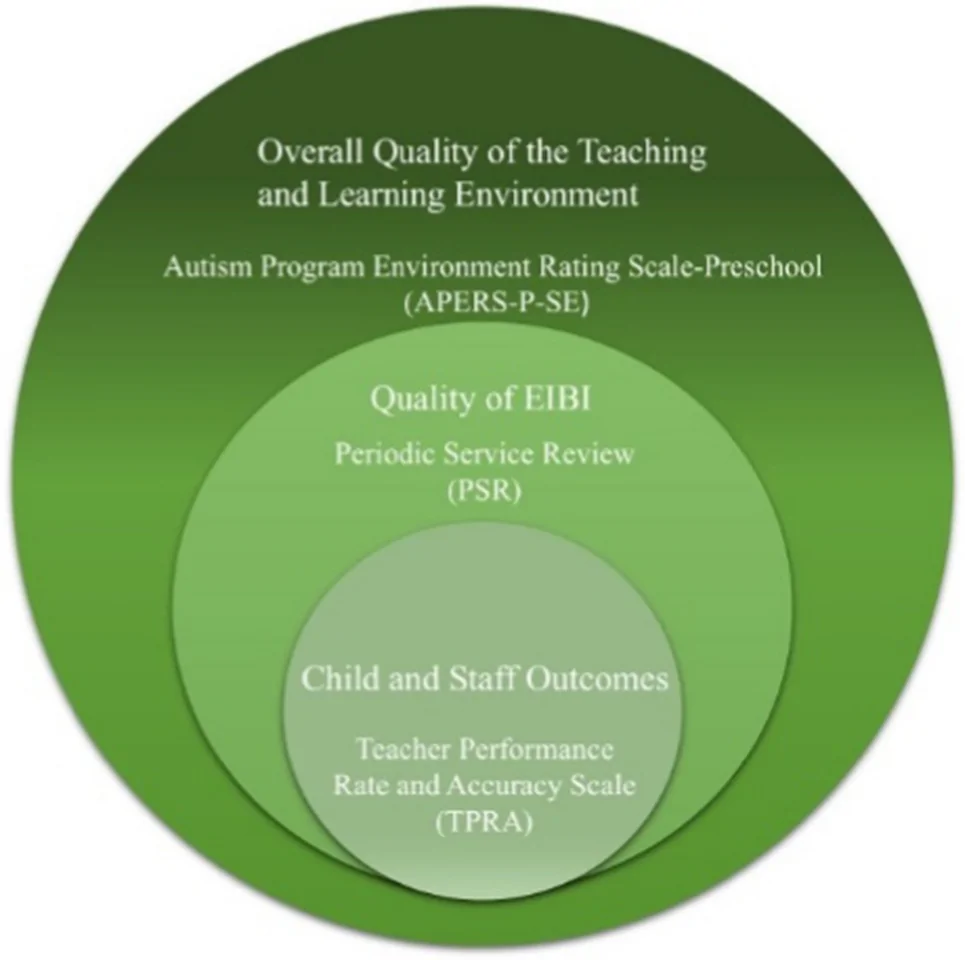
Complementarity of three dependent variables across levels

The APERS-P-SE (Bejnö et al., 2019), TPRA (Ross et al., 2005), and PSR (LaVigna et al., 1994) were conducted during baseline, the 17-week intervention period (not APERS-P-SE), post-intervention, and follow-up 10 weeks after the termination of the intervention. The social validity scale (SVS) was administered post-intervention. To ensure a comprehensive assessment, multiple measures were employed to evaluate the effects of the independent variable, with each measure complementing the others (Fig. 1). These measures targeted different but interconnected aspects of preschool quality. The APERS-P-SE and PSR assess distinct aspects of preschool quality. While the APERS-P-SE evaluates the overall quality of the teaching and learning environment, the PSR focuses specifically on the quality of the EIBI program. In contrast, the TPRA emphasizes operationalizing specific components across multiple domains within the APERS-P-SE and enhancing staff competencies essential to effectively implementing the EIBI program.

#### Autism Program Environment Rating Scale-Preschool-Sweden (APERS-P-SE)

The Autism Program Environment Rating Scale-Preschool/Elementary School (APERS-PE) is a validated assessment tool designed to evaluate inclusive practices by identifying strengths and weaknesses. It can serve as a foundation for action plans aimed at enhancing overall educational quality (Odom et al., 2018). In this study, we used a version of the APERS-PE adapted to the Swedish community context (APERS-P-SE), which has demonstrated high cross-cultural validity (Bejnö et al., 2019). Table 1 provides an overview of the content of APERS-P-SE, which consists of 56 subdomains unevenly distributed across 10 domains.

**Table 1:** Description of Autism Program Environment Rating Scale-Preschool-Sweden (APERS-P-SE)

	Domain	Subdomain
1	Learning environment	Safety, organization of learning environment, materials, visual schedules, transitions
2	Positive learning climate	Staff-student interaction, staff behaviors, promoting diversity
3	Assessment and IEP development	Assessing student progress, assessment process, IEP goals, transition planning
4	Curriculum and instruction	Classroom instruction
5	Communication	Communication rich environment, individualized communication instruction, responsiveness to student communication, communication systems
6	Social competence	Arranging opportunities, teaching and modeling, peer social networks
7	Personal independence and competence	Personal independence, self-management
8	Functional behavior (interfering and adaptive)	Proactive strategies, behavioral assessment, behavioral management, data collection
9	Family involvement	Teaming, communication, parent-teacher meetings
10	Teaming	Team training, team membership, team meetings, implementation

The table is derived from Bejnö et al. (2019)

Table 2 presents an example of one of the 56 items, item 21, from the Curriculum and Instruction domain. The numbers 1, 3, and 5 represent the rating scale. A score of 1 is assigned if any indicators under option 1 are observed and checked on the score sheet. A score of 2 is given if no indicators for 1 are checked, but at least one for 3 is marked. To receive a rating of 3 on item 19, it is sufficient for a key staff member, typically a special education teacher, to perform the tasks as expected. A score of 4 is assigned when all indicators for 3 are checked, along with at least one for 5. A score of 5 requires two or more staff members to collaboratively plan and implement interventions, ensuring that the child has opportunities to practice learned skills across various settings, with both checkboxes marked. It is important to note that higher scores correspond to a greater number of staff members actively engaging in task implementation.

**Table 2:**
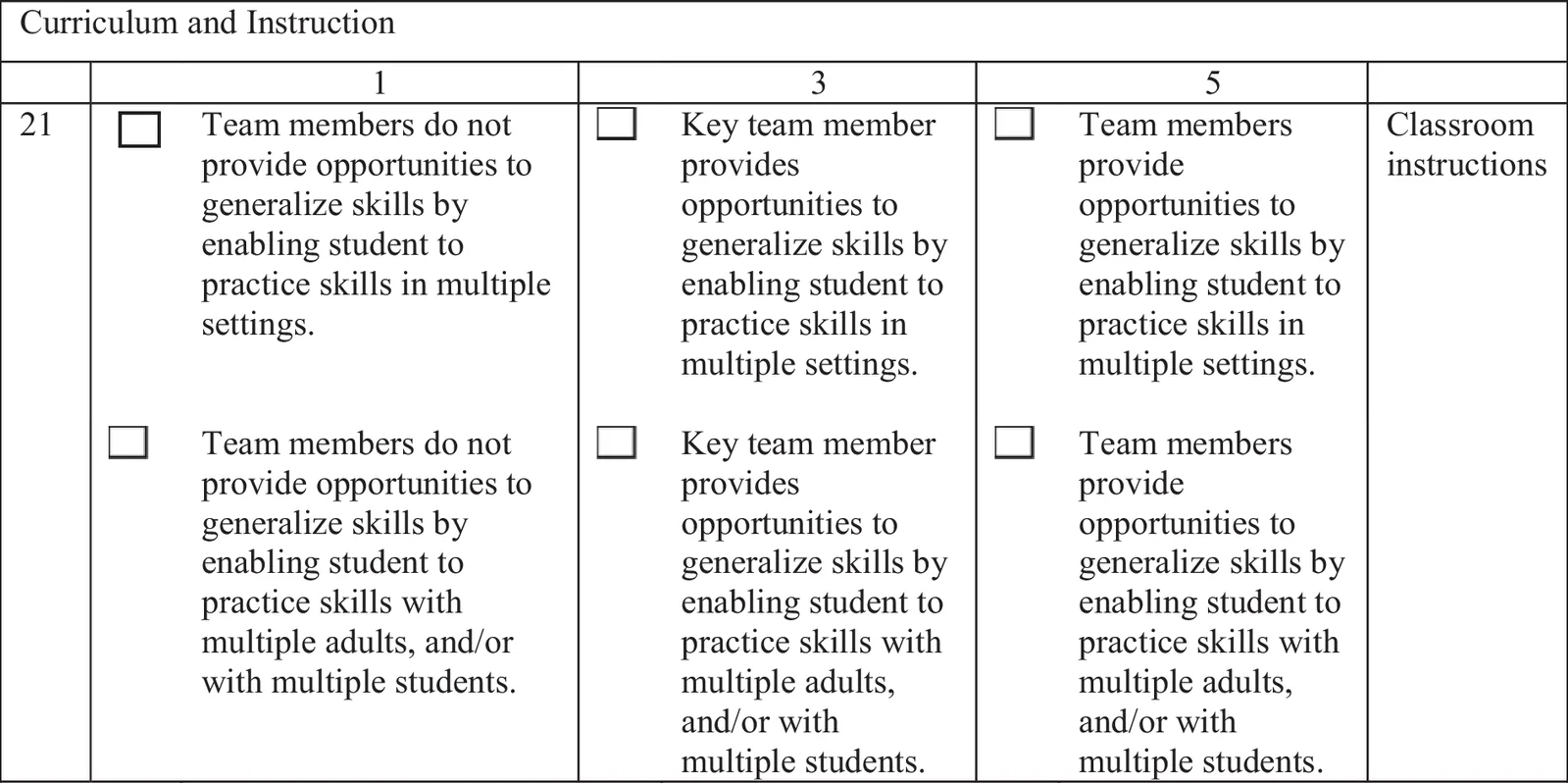
Example of a scoring rubric with behavior criteria for different scores (1–5) on the Likert scale in APERS-P-SE item 21 and corresponding behavior descriptions

During the APERS-P-SE observations, the staff performed a routine morning session of free play, mealtime, transitions, special education sessions, and sometimes circle time. The first author and sometimes an EIBI supervisor observed approximately 4 h. They moved around the classroom unit and noted events and the employee’s behavior to gather information to score the APERS-P-SE. In addition, we collected data on evocative situations during lunchtime. The observers provided no feedback to the participants during the observation period. Upon completion of the observation, interviews were conducted with the pedagogical leader of the unit and the parents, the documentation was reviewed, the results were graphed, and a report was written. APERS-P-SE is comprehensive, with each observation day, including interviews and scoring, taking approximately 7 h.

We administered APERS-P-SE twice to establish a baseline, then once at the posttest, and follow-up 10 weeks after the end of the intervention. The first author and an EIBI supervisor conducted Inter-rater reliability (IRR) on the APERS-P-SE in 50% of the sessions. IRR was assessed using a two-way mixed, consistency, average-measure Intra-Class Correlation (ICC) (McGraw & Wong, 1996) to determine the degree to which observers were consistent in recording APERS-P-SE. This ICC variant was chosen, among other reasons, because APERS-P-SE is a tool that uses Likert Scale scores, and the current study had designated observers. The ICC measures the magnitude of agreement rather than simply agreement or disagreement (e.g., assessing consistency in multiple observations of staff behaviors across different sessions). The resulting ICC was in an excellent range (Cicchetti, 1994) during baseline distributed as follows: Preschool A = .94; Preschool B = .80; and Preschool C = .91. The posttest results showed excellent agreement distributed as follows: Preschool A = .96; Preschool B = .91; and Preschool C = .98. In addition, the percentage of agreement was calculated by exact agreement on each of the 56 statements in APERS-P-SE, with the number of agreed statements divided by the total number of statements and multiplied by 100. Table 3 provides the APERS-P-SE percentage of agreement and ICC scores for the participating preschools during baseline and posttest.

**Table 3:** Summary of inter-rater reliability of Autism Program Environment Rating Scale-Preschool-Sweden (APERS-P-SE)

Participant	Baseline		Posttest		
	%	ICC	%	ICC	p
Preschool A	82	.94	76	.96	.000
Preschool B	69	.80	78	.91	.000
Preschool C	82	.91	85	.98	.000

% = percentage of agreement; *ICC* Intra-class correlation.

APERS-P-SE uses a Likert scale, and its 56 items are broadly formulated without distinct response definitions, making it difficult to achieve the recommended percentage of 80. However, the ICC results (excellent agreement) indicate how closely the observers’ ratings align, providing a more nuanced picture of the ratings’ reliability rather than merely making a binary calculation through percentage agreement.

#### Periodic Service Review (PSR)

The PSR is a quality improvement system designed to help staff assess service quality and identify specific areas for enhancement. By implementing a systematic approach, PSR establishes a framework for continuous quality improvement through regular evaluation cycles and data-driven decision-making (LaVigna et al., 1994). This structured methodology supports service providers in maintaining high standards of care while simultaneously fostering professional development and program refinement. Research suggests that staff generally perceive PSR as a valuable quality monitoring tool (Lowe et al., 2010).

On the basis of the principles of the PSR, we developed a checklist to assess the quality of the EIBI (LaVigna et al., 1994). The development of the PSRs was based on existing frameworks and adapted to the preschools participating in this study. To ensure consistency, the PSRs were standardized and implemented uniformly across all preschools. Table 4 provides an overview of the 18 true–false statements in the PSR.

**Table 4:** Overview of 18 true–false statements utilized in the periodic service review (PSR)

#	True–False statement
1	The child received 20 h of weekly training on objectives through one-on-one instruction, group settings, or classroom activities
2	A minimum of 90% of the scheduled sessions were successfully completed
3	At least two staff members in the preschool provided intervention
4	Biweekly team meetings were held in the preschool
5	Practical tasks (e.g., preparing materials, filming) were completed as agreed
6	The program overview was updated following each meeting
7	Program descriptions were regularly updated in the program binder
8	The child had individualized objectives within a small group of peers
9	The child had objectives implemented outdoors and across various spaces within the preschool
10	Scheduled consultations with parents were conducted to support daily follow-up at home
11	Registrations were completed as agreed, including data on eating, sleeping, and problem behavior
12	Overall progression was assessed every three months during the first six months and every six months thereafter
13	The PSR was included as part of the evaluation process
14	Self-assessment of training skills was conducted
15	Assessment of Skills (ASK) and Assessment of Basic Language and Learning Skills-Revised (ABLLS-R®) data were included in the evaluation
16	Annual reports were developed in accordance with municipal guidelines
17	All members of the training team had completed EIBI courses
18	All team members were assessed on their teaching skills

The first author and the special educator scored the quality standard once a month, taking approximately 20 min each time. Whenever a disagreement occurred, the first author and special education staff discussed the statement until they reached an agreement. Hours spent with the EIBI program (20 h per week) were recorded as average based on the hours completed in the last month. The other 17 statements were scored as true or false, depending on whether the task was completed. The percentage was calculated by dividing the total score by the maximum possible score. The PSR was administered twice during baseline, four times during the intervention period, and once at follow-up conducted 10 weeks after the intervention ended. 

#### Teacher Performance Rate and Accuracy (TPRA)

The Teacher Performance Rate and Accuracy (TPRA) is a system designed to measure the implementation of evocative situations and has been shown to be a feasible tool for assessing treatment integrity (Hranchuk & Williams, 2021). TPRA provides data on the behavior of the child and the employee, offering valuable insight that can be utilized to evaluate training effectiveness and continuously adjust behavior.

The rate and accuracy of staff implementation of evocative situations were assessed weekly during baseline using the TPRA (four times for preschool A, three times for preschool B, and five times for preschool C). The first TPRA assessment was conducted within a week of completing the first part of the intervention (i.e., the 4-h training workshop), followed by eight assessments every two weeks on a different day than the biweekly coaching sessions. The observation period, including transportation, took approximately 50 min. No feedback was given to the staff during or after the observation. Finally, an assessment was conducted at follow-up, 10 weeks after the end of the intervention.

The observation period started once the child was seated and ended when 5 min had elapsed without a request for food or drink or before the child indicated that mealtime was finished. Hence, the duration of the observation varied, ranging from 12 to 40 min, and evocative situation data were presented as rate per minute. The children’s semi-preferred foods (e.g., fish sticks, apples, bell peppers, and fish cakes) and beverages were offered during meals, rather than highly preferred foods like fast food and chips. Observers were located a few meters behind the participants to avoid disturbing the children and staff, but close enough to observe the employee and the child. Observers collected data on both staff implementation and children’s responses during each evocative situation trial.

A plus ( +) or minus ( −) was written on the TPRA form for antecedent, child response, and consequence. The antecedent was scored correctly when the employee established the child’s attention and provided clear, concise, and specific instruction. The child’s behavior was correct if the response occurred within 3 s and corresponded to the instruction (i.e., if the employee said, “Give me milk,” and the child handed milk) or the child manded (a verbal request or picture exchange) for a preferred item. A minus ( −) in the consequence column indicated an incorrect consequence based on the child's response. For example, if the employee provided the antecedent, “Give me milk,” and the child handed bread, the participant responded, “Thank you, nice job!”. In this case, the child’s response was also recorded as a minus ( −). The antecedent and consequence had to be recorded with a plus ( +) to be considered a correct evocative situation.

We performed an IRR analysis using Cohen’s Kappa (κ) as a reliability index to assess the degree to which observers consistently scored the evocative situations. Cohen’s Kappa (κ) measures the observed level of agreement between coders and adjusts for the level of agreement expected by chance (Hallgren, 2012). The baseline results showed perfect agreement, while there was overall substantial agreement during the intervention period, distributed as follows: Preschool A = 0.77, Preschool B = 1.00, and Preschool C = 0.80. In addition, the percentage of agreement was calculated trial by trial with the number of trials in agreement divided by the total number of trials and multiplied by 100. At baseline, there was 100% agreement on all measurements. During the intervention, inter-rater reliability scores for preschool A were 87% (range 76–93), preschool B 100%, and preschool C 93% (range 90–100). The first author assessed evocative situations, while the child’s EIBI supervisor collected interobserver data for 30% of the baseline and intervention sessions. Table 5 shows the inter-rater reliability scores for the evocative situations in the participating preschools during baseline and intervention.

**Table 5:** Inter-rater reliability of the evocative situations for the participating preschools

Participant	Baseline	Intervention		
	%	% (range)	κ	*p*
Preschool A	100	85 (76–93)	.77	.000
Preschool B	100	100	1.00	
Preschool C	100	93 (90–100)	.80	.000

% = percentage of agreement; *κ* Cohen’s Kappa

#### Social Validity

On the basis of Bejnö et al. (2021), we developed a social validity scale (SVS) for the current study, scored on a 7-point Likert scale (0 = totally disagree, and 7 = totally agree). It was designed for preschool staff to assess the relevance and meaningfulness of the objectives based on APERS-P-SE targeted within the study, the methods used, the feedback provided by the research team, the content of the in-service training and coaching, and the future usefulness of the knowledge and skills acquired when working with other children. See Table 6 for an overview of the statements in the SVS. The staff independently reviewed the questionnaire and rated each statement post-intervention.

**Table 6:** Results of the social validity scale of nine employees in the three participating preschools

		Preschool A		Preschool B			Preschool C				
	Questions/employees (N)	N1	N2	N3	N4	N5	N6	N7	N8	N9	Mean
1	The training and supervision were relevant to the inclusion work in the preschool	7	7	7	6	6	7	7	6	6	6.50
2	The goals we worked on were meaningful and relevant	7	7	7	6	6	7	6	6	7	6.50
3	The methods we used were helpful in working with children with special educational needs	5	6	7	6	6	7	7	7	7	6.40
4	I have gained new knowledge and learned new skills that make me a better teacher	6	6	7	6	7	6	7	7	6	6.40
5	During the project period, the child has learned new skills and is more included in the children's group	3	2	7	3	4	7	7	5	6	4.80
6	I would recommend other preschools to do this intervention	7	7	7	6	7	7	7	7	7	6.80
	Total mean										6.23

Likert scale (0–7), where 0 is “I completely disagree,” and 7 is “I completely agree”; *N* participants

## Results

### Autism Program Environment Rating Scale-Preschool-Sweden (APERS-P-SE)

**Fig. 2:**
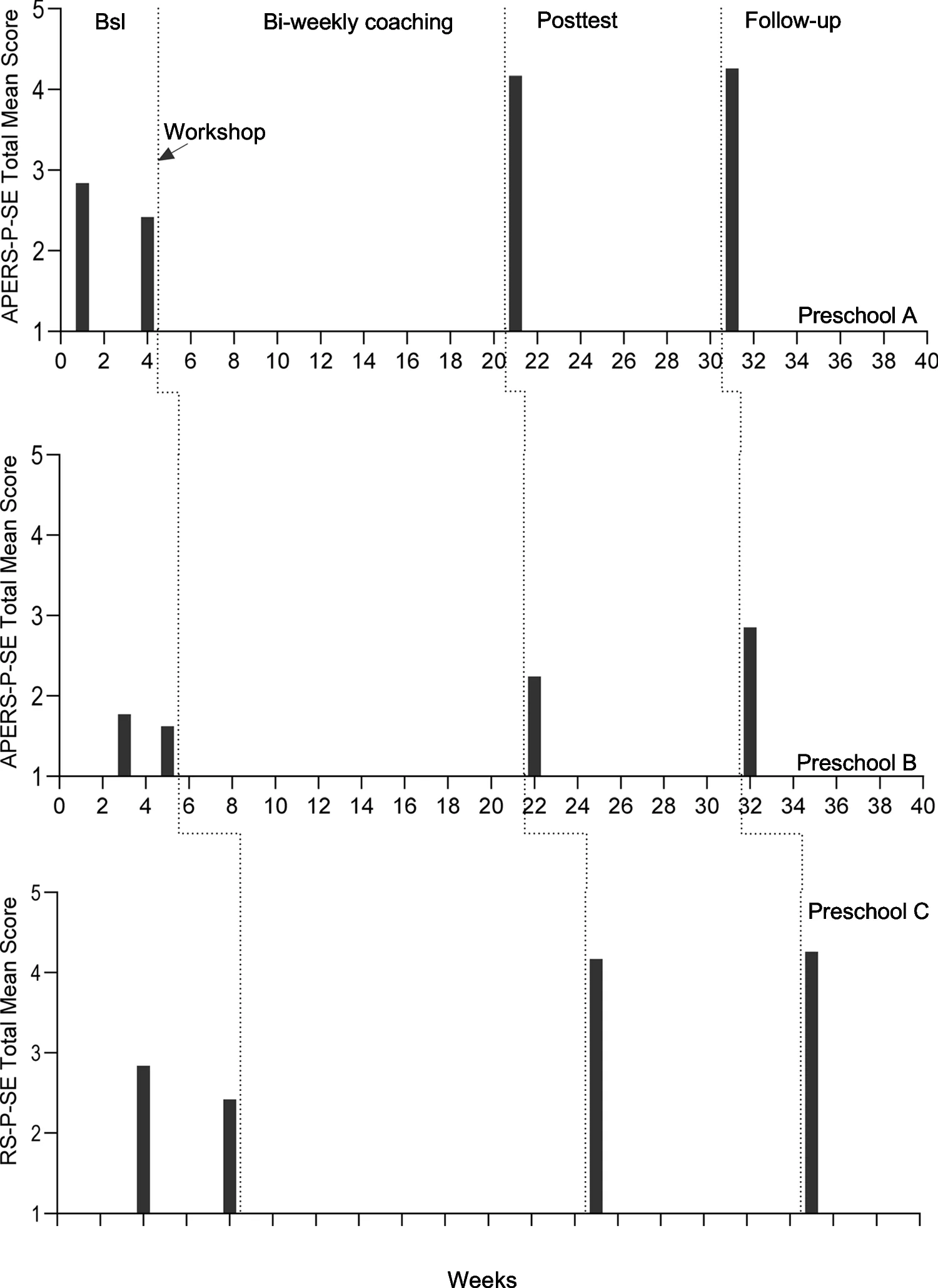
Changes in APERS-P-SE total mean score. *Note. APERS-P-SE* Autism Program Environment Preschool Sweden; *Bsl* Baseline

Figure 2 shows the APERS-P-SE total mean score during the pretest, post-intervention, and follow-up for the three participating preschools. For all three preschools, an improvement occurred after intervention (i.e., the in-service training and eight biweekly coaching sessions) and was maintained at follow-up.

**Fig. 3:**
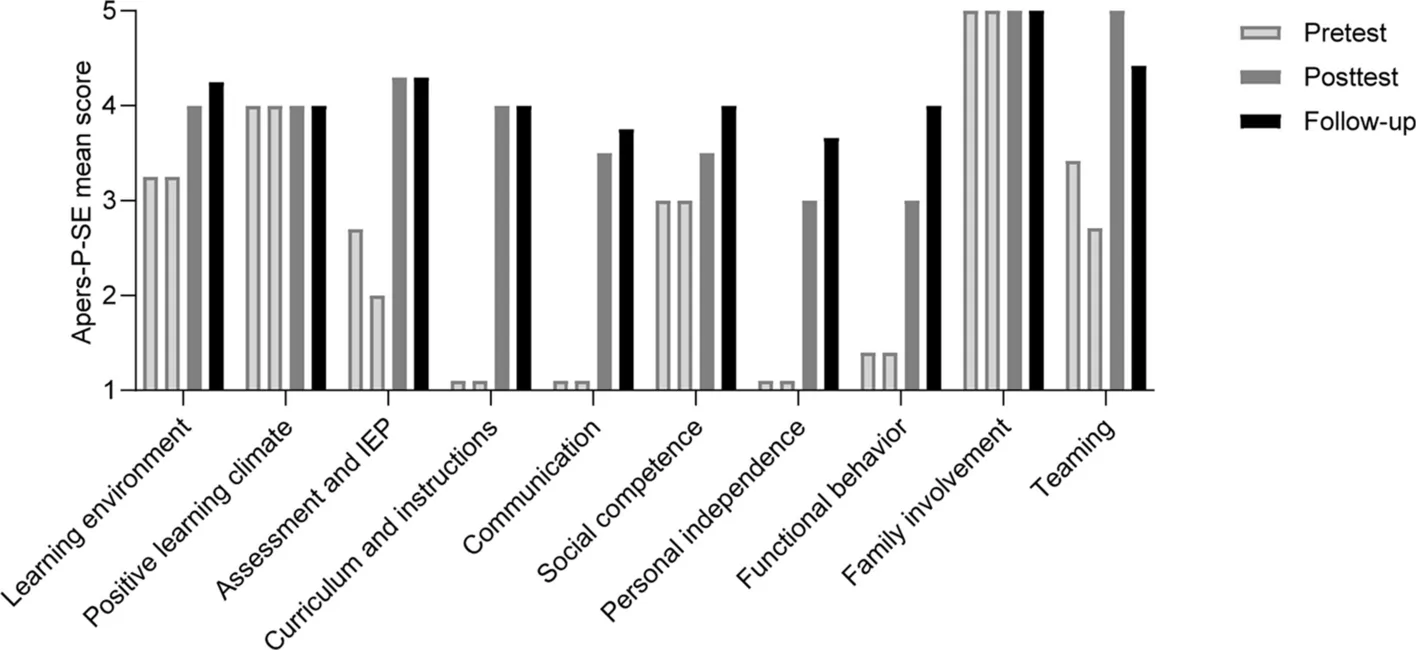
Changes in APERS-P-SE domains in preschool A. *Note. APERS-P-SE* Autism Program Environment Preschool Sweden

**Fig. 4:**
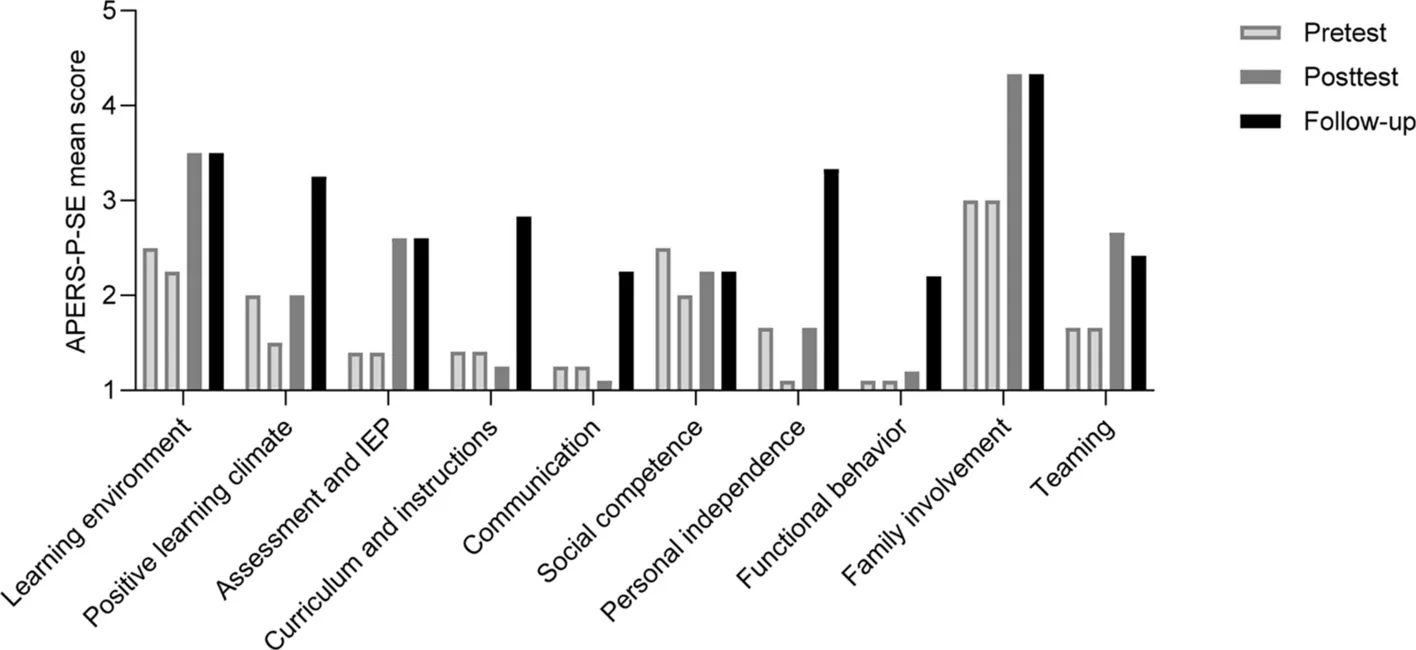
Changes in APERS-P-SE domains in preschool B. *Note. APERS-P-SE* Autism Program Environment Preschool Sweden

**Fig. 5:**
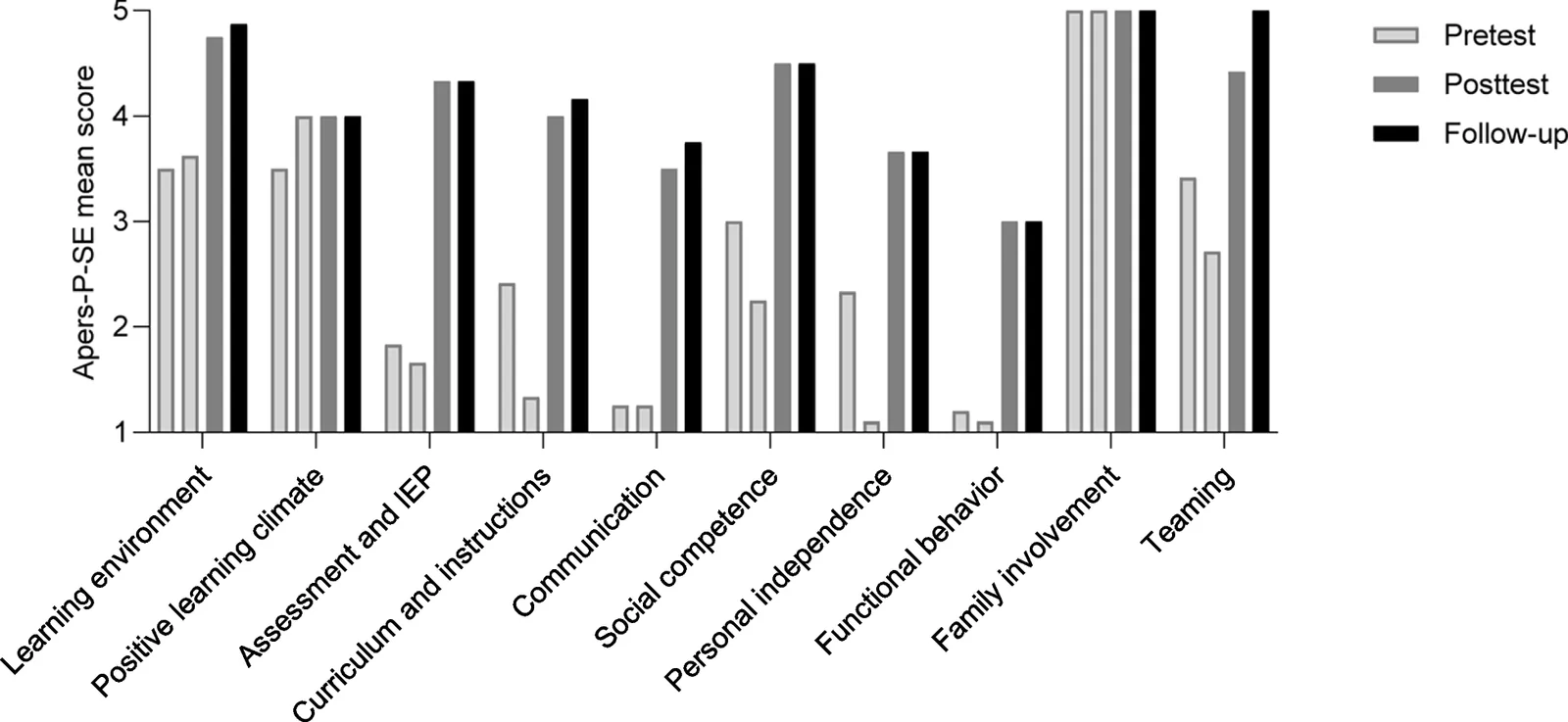
Changes in APERS-P-SE domains in preschool C. *Note. APERS-P-SE* Autism Program Environment Preschool Sweden

Figures 3, 4, 5 show scores on each of the 10 APERS-P-SE domains during pretest, post-intervention, and follow-up for preschools A, B, and C, respectively. For preschool A, there was an improvement in eight out of 10 domains between baseline and intervention. The improvement was maintained at follow-up. The Family Involvement and Positive Learning Climate domains remained stable across assessments; the former was due to a ceiling effect. In Preschool B, the quality of the APERS-P-SE across nine domains improved from baseline to follow-up. Interestingly, improvement in four domains was only observed at follow-up and not at posttest. For preschool C, there was improvement in eight out of 10 domains between baseline and intervention, and the improvement was maintained at follow-up.

### Periodic Service Review (PSR)

**Fig. 6:**
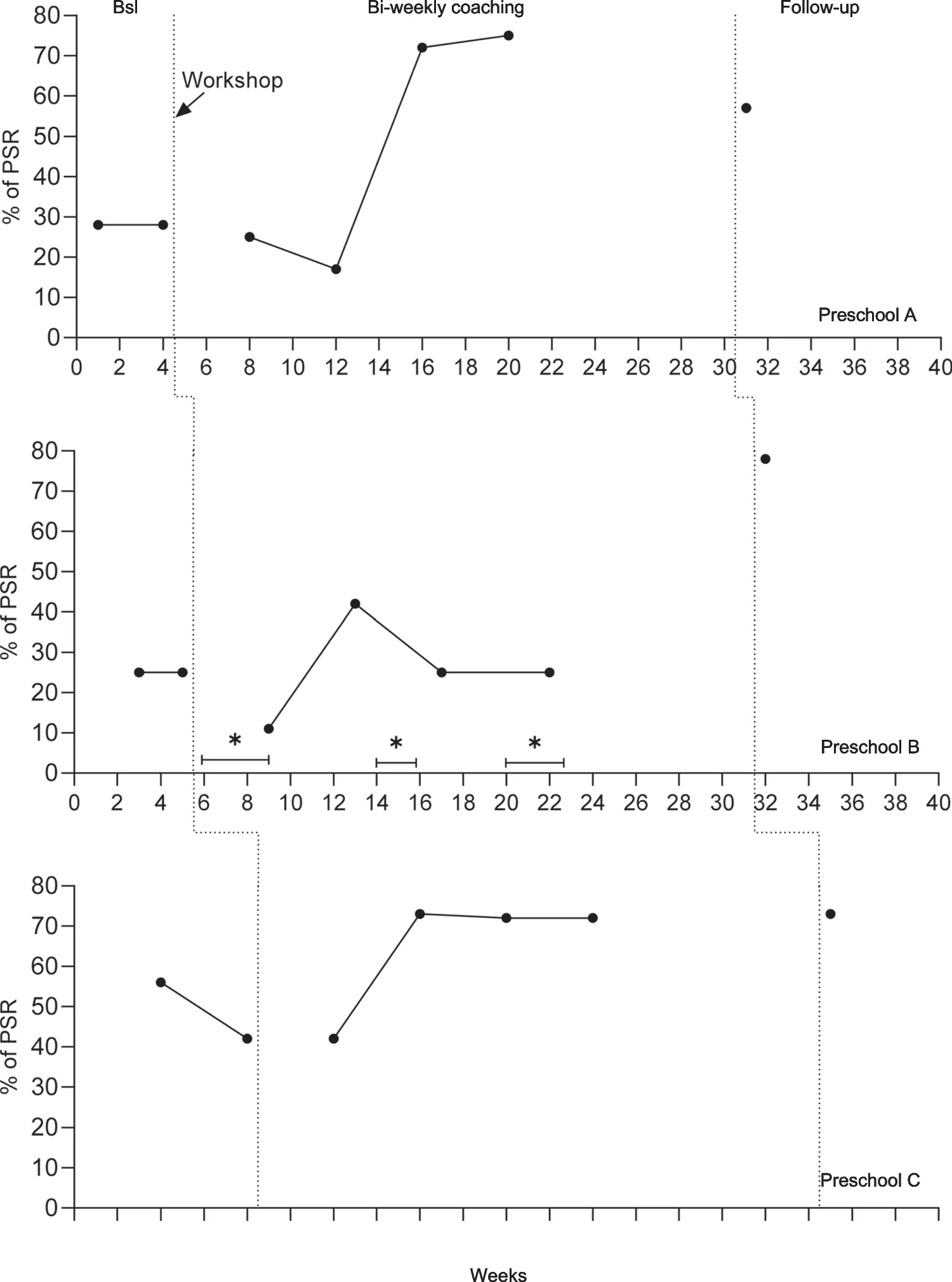
Changes in EIBI quality in participating preschools. *Note. EIBI* Early intensive behavioral intervention; *Bsl* Baseline; *PSR* Periodic service review; Star with an underline indicates the special education teacher's sick leave at preschool B

Figure 6 shows the quality of EIBI during baseline, intervention, and follow-up for the participating preschools. Compared to the baseline, the in-service training workshop did not result in any notable changes in the quality of EIBI for any of the three preschools. However, for preschool A and C, the quality of EIBI improved after 16 weeks of biweekly coaching. For preschool B, the quality of EIBI did not improve until follow-up. For preschool A, the EIBI quality experienced a slight decline at follow-up, although it remained substantially higher than the baseline level. Preschool C, in contrast, maintained the improved EIBI quality during the follow-up assessment.

### Teacher Performance Rate and Accuracy (TPRA)

**Fig. 7:**
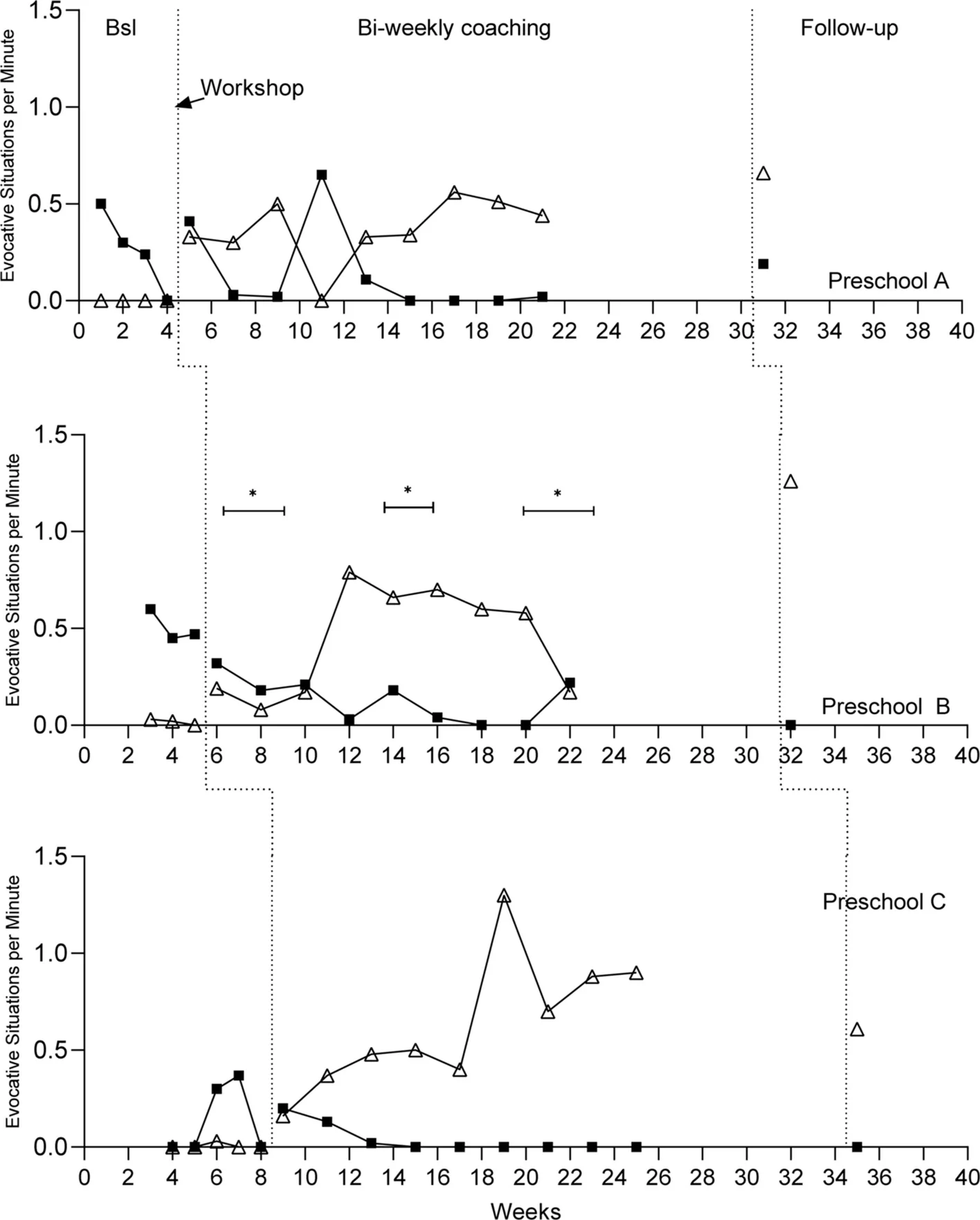
Correct and incorrect staff implementation of evocative situations during lunch. *Note. Bsl* Baseline; Star with an underline indicates the special education teacher’s sick leave; Coaching sessions occurred one week after each data point during the biweekly coaching period; Δ Correct implementation of evocative situations; ■ Incorrect implementation of evocative situations

Figure 7 illustrates the rate of correct and incorrect evocative situations per minute across the three participating preschools during baseline, intervention, and follow-up. Preschool A’s rate of correct evocative situations during baseline was 0. However, immediately following the in-service training workshop, the rate of correct evocative situations increased to 0.33 per minute. In nine biweekly assessments, the rate of correct evocative situations ranged from 0.33 to 0.56 per minute, except for week 11, where the rate of correct evocative situations was 0. During the follow-up assessment, conducted 10 weeks after the end of the intervention, the rate of correct evocative situations was 0.66. During the baseline phase, the rate of incorrect evocative situations ranged from 0.5 to 0 evocative situations per minute. Immediately following the workshop, the rate of incorrect evocative situations was 0.4. However, across the biweekly assessments during the intervention phase, the rate of incorrect evocative situations ranged from 0 to 0.65 per minute, peaking at week 11. In the follow-up assessment, the rate of incorrect evocative situations decreased to 0.19.

In preschool B, the rate of evocative situations was close to 0 during the first two baseline sessions and 0 at the third and final baseline sessions. Immediately following the in-service training workshop, the rate of correct evocative situations per minute increased to 0.19. In nine biweekly assessments, the rate of correct evocative situations ranged from 0.08 to 0.79 per minute. At the follow-up, conducted 10 weeks after the intervention’s end, the correct evocative situations rate was 1.26. The rate of incorrect evocative situations during the baseline phase ranged from 0.47 to 0.60. Across nine biweekly assessments during the intervention phase, the rate of incorrect evocative situations decreased, ranging from 0.32 to 0. No incorrect evocative situations were observed during the follow-up phase.

In preschool C, the rate of correct evocative situations was at or near 0 during the five baseline sessions. Immediately following the in-service training workshop, the rate of correct evocative situations per minute increased to 0.16. Across nine biweekly assessments during the intervention phase, the rate of correct evocative situations ranged from 0.16 to 1.30 per minute. During the follow-up assessment, conducted 10 weeks after the intervention, the rate of correct evocative situations was 0.61. The rate of incorrect evocative situations during baseline ranged from 0.37 to 0. Across nine biweekly assessments during the intervention phase, the rate of incorrect evocative situations decreased, ranging from 0.13 to 0. No incorrect evocative situations were observed during the follow-up phase.

**Fig. 8:**
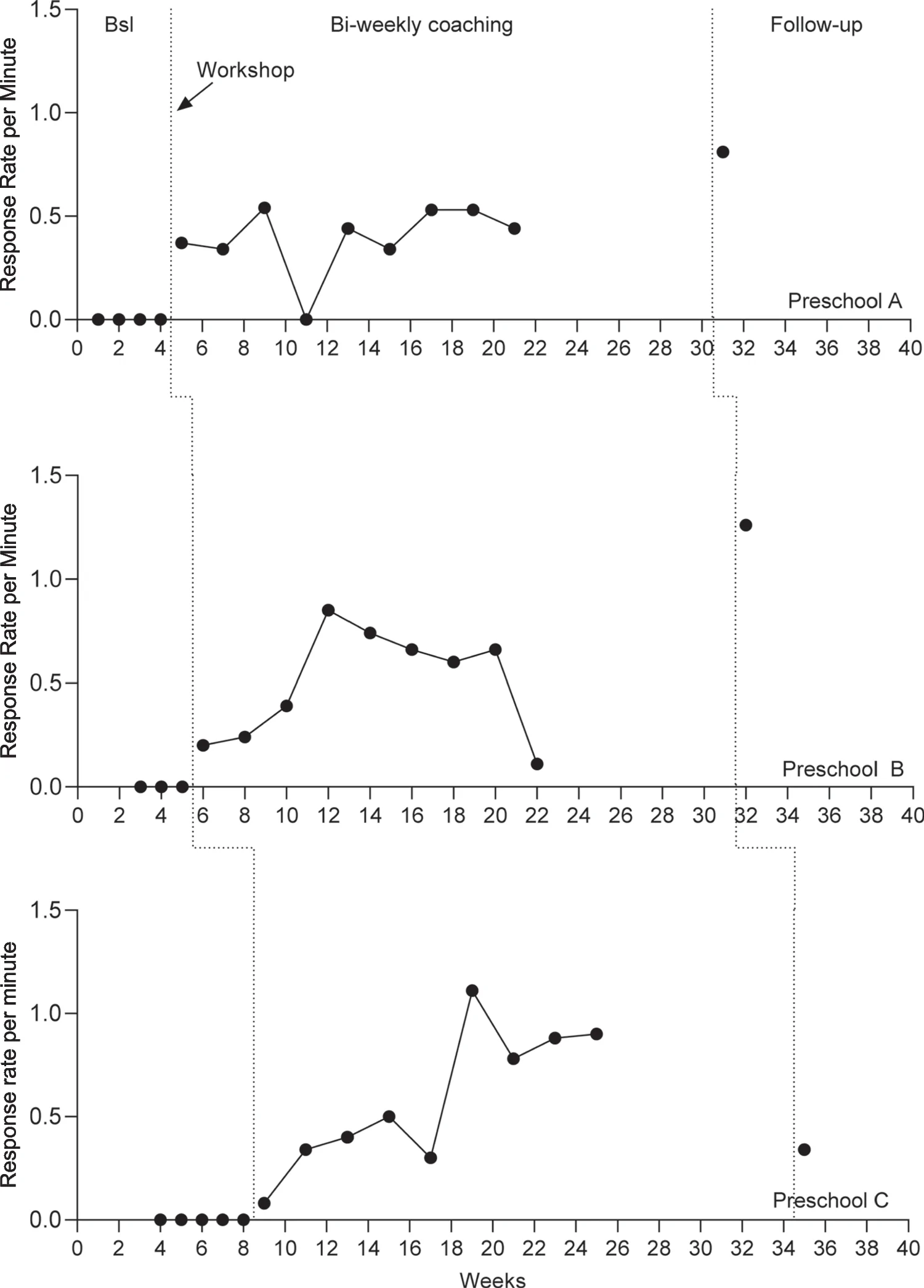
Changes in the participating children’s response rates per minute during lunch. *Note. Bsl* Baseline; Each biweekly coaching session was scheduled one week after the corresponding data point

Figure 8 illustrates the response rates per minute of the participating children (i.e., mand and listener skills) during lunch across baseline, intervention, and follow-up phases. None of the children exhibited mand or listener skills during the baseline phase. Following the staff’s in-service training workshop, the child in preschool A immediately increased the response rate to 0.37 responses per minute. During the intervention period, the response rate ranged between 0 and 0.54 responses per minute. At the follow-up assessment, the response rate increased to 0.81 responses per minute. At the start of the intervention period, the child in preschool B showed an initial response rate of 0.20 responses per minute. Throughout the intervention, the response rate ranged between 0.11 and 0.85 responses per minute. At the follow-up assessment, the response rate increased to 1.26 responses per minute. For the child in preschool C, the first measurement after baseline showed a response rate of 0.08 responses per minute. Throughout the intervention period, the response rate ranged from 0.08 to 0.90 responses per minute. At follow-up, the response rate decreased to 0.34 responses per minute.

Effect sizes for the evocative situations were estimated by the web application scdhlm (Pustejovsky et al., 2023). The between-case standardized mean difference (BC-SMD) estimation is based on Pustejovsky et al. (2014) and Hedges et al. (2013) and yields SMD comparable to Cohen’s d, with a small sample correction analogous to Hedges’s g (Pustejovsky et al., 2014, p. 368). The estimation was employed using restricted maximum likelihood, with fixed and random level effect for the baseline data and fixed effect change in level analysis for the treatment phase data, including AR1 analysis for autocorrelated data and constant session level variance (Valentine et al., 2016). The estimated effect size on evocative situations was large (i.e., above .8), g = 1.13, 95% CI [.19, 2.06], and considered statistically significant at the .05 level, as the confidence interval did not cross 0. The estimated effect size yields the same picture as the visual analysis in Fig. 7.

### Social Validity

The results of the social validity assessment are shown in Table 4. The staff rated five out of six questions to be close to the maximum score, with M = 6.5 (range 6.4–6.8). For question 5, participant responses varied, resulting in a mean score of 4.8.

## Discussion

This study examined the impact of a unit-wide in-service training package in three inclusive preschool units. The training included a theoretical session, goal selection based on APERS-P-SE, BST, and biweekly coaching, aiming to enhance the quality of the EIBI program for the target child within the unit, as well as the overall teaching and learning environment. The findings indicate improvements in the overall teaching and learning environment, as well as EIBI quality in two preschool units. As part of the effort to improve the general learning environment, the unit staff were trained to provide evocative situations during lunch. The increased staff proficiency in accurately providing evocative situations during lunch increased in all three preschools. Additionally, the participating children with special needs demonstrated increased mand and listening skills during lunchtime. These results indicate that the staff training program and biweekly coaching had a positive impact on staff behavior in preschool A and C, whereas the effects were more modest in preschool B. However, variations in effectiveness raise important questions regarding the intervention’s impact and contextual factors. In the following discussion, we explore potential explanations for these differences, evaluate the impact of the training package components, and outline future research directions.

The first research question examines the overall program quality and EIBI implementation in three inclusive preschools. A key aspect of the evaluation is analyzing the various components of the training package. The first component, theoretical in-service training, followed a stepwise approach, in which PowerPoint-supported lectures interspersed with questions, participant reflections, and plenary discussions provided a theoretical foundation for subsequent practical components. This structure was particularly important, given that the majority of staff had limited experience with behavior-analytic work. These knowledge gaps, consistent with previously identified barriers (Eldevik et al., 2019; Layden et al., 2024; Roll-Pettersson et al., 2016), were also reflected in the APERS-P-SE findings. Research suggests that although lectures may increase knowledge, they often fail to promote the transfer of that theoretical knowledge to practical application in real-world settings (Schachter et al., 2019). In addition, lectures with many participants often fail to account for the need to tailor instruction to individual and contextual factors (Schachter et al., 2019). This study sought to address this limitation by integrating APERS-P-SE assessments to inform content development, thereby increasing the training’s applicability. However, no pre- and post-lecture assessments were conducted, making it difficult to determine the exact impact of the lectures on participants’ knowledge. While foundational knowledge is necessary for effective implementation, theoretical training alone is unlikely to produce meaningful behavior change without opportunities for coaching and feedback (Schachter et al., 2019).

The second training component, goal setting, utilized the APERS-P-SE assessment as an objective framework for identifying areas in need of improvement. Consistent with previous research demonstrating its effectiveness, APERS-P-SE guided intervention planning by targeting both overall enhancements in the teaching and learning environment and individualized goals for staff (Bejnö et al., 2021). Rather than imposing predetermined objectives, goal setting was informed by assessment results, allowing staff to participate in the selection process and ensuring a data-driven foundation for intervention (McLucas et al., 2024). Staff’s active involvement in selecting and prioritizing development areas may have contributed to enhancing the perceived relevance of the training, as supported by the results of the SVS questionnaire (item 2), which indicate that participants found the goals meaningful and relevant. Importantly, goal setting is a foundational element in effective coaching, as it provides a structured basis for feedback and skill development (Elek et al., 2019; McLucas et al., 2024). However, isolating the effects of goal setting alone is challenging, as this component is rarely implemented without concurrent coaching and feedback (McLucas et al., 2024).

APERS-P-SE results for preschools A and C showed improvements in eight of ten domains, with gains maintained at follow-up. Several domains exceeded a score of 4, indicating that multiple staff members, beyond the special educator, successfully implemented routines and activities that promote children’s development. These findings suggest that, following the intervention, children experienced an improved educational environment throughout the day rather than only when the special educator was present. This improvement is a promising indicator of enhanced inclusion in preschools A and C. Similar results were reported by Bejnö et al. (2021), who found that systematic assessment using APERS-P-SE, combined with targeted interventions and coaching, significantly improved the preschool learning environment for children with ASD. While the in-service training package appears to have had the intended effect, it remains unclear which component contributed the most. Future research should aim to identify the effects of the individual components, such as the theoretical session, goal selection based on APERS-P-SE, BST, and biweekly coaching, to determine which elements produce the greatest impact.

Preschool B, which had substantially lower pretest scores reflecting lower program quality before the intervention, showed significantly less progress in posttest and follow-up assessments. These findings suggest that preschools with initially low program quality may require more intensive and sustained support, including strong leadership involvement, to achieve meaningful improvements. This aligns with prior research indicating that lower baseline quality in early childhood settings is associated with greater challenges in implementing evidence-based practices (Fixsen et al., 2009; Odom et al., 2020). Although preschool B started with lower program quality, improvements were observed in five domains immediately after the intervention. Additionally, four domains, Positive Learning Climate, Curriculum and Instruction, Communication, and Personal Independence, showed delayed improvements, emerging only at follow-up. It is noteworthy that only the Family Involvement domain received a score above 4 in preschool B. Scores below this threshold indicate that few staff members actively contributed to implementing everyday routines and activities that support children’s development.

Several factors may explain the results in preschool B. Six of eight coaching sessions were disrupted due to staff illnesses, including the special educator, and a shortage of substitutes during the COVID-19 pandemic. As a result, sessions were conducted with available staff, but inconsistency in participants likely contributed to the lack of progress observed in the posttest. Given the improvements observed in five domains following the biweekly coaching sessions, it is reasonable to suggest that the in-service training package may have had some impact on staff behavior and APERS-P-SE outcomes. The results from preschool B highlight the need for stronger leadership support, including more direct and continuous guidance for staff, as well as targeted coaching and supervision for leaders. Such support is critical not only for facilitating and sustaining the implementation of EBPs but also for advancing the overall quality of the teaching and learning environment, which is fundamental to children’s development. To strengthen the impact of EBP implementation in preschools with initially lower program quality, future research should examine effective strategies for enhancing leadership involvement. Given that sustained practice change often requires ongoing feedback, studies should explore how leadership support, both in terms of direct staff supervision and leaders’ own training in instructional leadership, can facilitate long-term adherence to evidence-based practices.

As part of research question 1 (How does theoretical in-service training, including goal setting and biweekly coaching, influence the quality of EIBI and overall program quality in three inclusive preschools, as measured by the APERS-P-SE, and the PSR?), we aimed to evaluate the impact of in-service training and biweekly coaching on the quality of EIBI. Several factors may explain the initially low EIBI quality across all preschools, including insufficient intensity and the absence of a trained EIBI team within the unit. Although children were expected to receive 20 h of EIBI per week, actual implementation fell short, with preschool A averaging 5 h, preschool B 4 h, and preschool C 6 h per week. Relying exclusively on the special educator to implement EIBI limited treatment consistency, reduced opportunities for generalization, and compromised service delivery during periods of absence. A trained team within the unit is essential for sustaining implementation and addressing common barriers, such as limited competence in ASD and EBPs, insufficient collaboration among key stakeholders, and difficulties in maintaining adequate intervention intensity and ensuring sufficient learning opportunities.

The theoretical in-service training workshop alone did not lead to immediate improvements in EIBI quality in any preschool. However, gains were observed in preschools A and C during the biweekly coaching sessions. While these improvements remained stable in preschool C, preschool A experienced a decline at follow-up. This pattern indicates that coaching and supervision may have played an important role in facilitating the observed improvements. The decline in preschool A at follow-up may be partially attributed to the child’s 10-day vacation shortly before the assessment, which reduced training intensity in the weeks leading up to the EIBI quality measurement. However, it is also possible that the level of support provided was insufficient to sustain lasting change. A subsequent follow-up assessment could have offered further insight, but after the summer break, the child transitioned to a different unit with new staff members, making continued evaluation impossible.

While preschools A and C established EIBI teams and increased weekly instructional hours during the intervention period, preschool B was unable to do so. Staggered sick leaves among regular staff, combined with frequent use of substitutes, prevented the establishment of a stable EIBI team. Periodic absences of the special educator further limited coaching opportunities and direct implementation, resulting in the child receiving less than the targeted 20 h of instruction per week. As a result, the quality of EIBI in preschool B remained compromised until follow-up. Figure 6 illustrates periods of the support teacher’s absenteeism throughout the intervention. Notably, she was present during the weeks leading up to the EIBI quality measurement in week 13 and consistently attended work for the eight weeks preceding the follow-up assessment. Her presence before the EIBI quality measurement in week 13 coincided with the highest recorded scores compared to baseline and subsequent EIBI quality measurements. A similar pattern was observed at follow-up, with increased scores corresponding to her stable attendance in the preceding weeks. This trend may reflect the absence of an EIBI team within the unit, resulting in a lack of instructional continuity when the support teacher was absent. It is important to note that scores reflect EIBI quality over the preceding four weeks. These findings underscore the importance of having an intact staff group present and distributing targeted work among several preschool employees rather than just the special education teacher. Involving the entire staff group when implementing EBPs in preschool settings may help mitigate systemic vulnerabilities, support the sustainability of high-quality special educational services, and promote a more consistent and inclusive teaching and learning environment for all children (Fixsen et al., 2009; Odom, 2000).

Regarding the second research question on preschool staff implementing evocative situations during lunch, we observed an immediate increase in the accurate use of evocative situations following the BST workshop, along with a reduction in incorrect implementations across all preschools. These results suggest that the in-service training workshop incorporating BST may have positively influenced the staff’s accurate implementation of evocative situations, aligning with previous research on the effectiveness of BST in improving staff performance (Courtemanche et al., 2021; Nigro-Bruzzi & Sturmey, 2010; Sarokoff & Sturmey, 2004). Improvements were observed throughout the intervention period. However, preschool B showed a decline in correct implementation at the final measurement point, when no further coaching was provided due to the conclusion of the project. In preschool A, we observed a notable decline in the correct implementation of evocative situations in week 11, coupled with a rise in incorrect evocative situations. Following additional coaching, improvements were again observed. The progression seen in preschool A during the intervention period with biweekly coaching, as well as the feedback and problem-solving implemented after week 11, suggests that coaching and supervision played a crucial role in staff members’ accurate use of evocative situations. This underscores that merely acquiring knowledge and skills through in-service training and BST does not automatically equip staff with the analytical skills or clinical judgment necessary for sustainable implementation. Instead, behavioral assessment, performance feedback, and ongoing coaching over an extended period are considered essential components for effective skill acquisition and maintenance (Kraft et al., 2018; Novak et al., 2019).

Determining which specific BST component contributed to staff acquiring accurate use of evocative situations is challenging. Previous research suggests that written instructions, including protocols, are insufficient for effectively developing staff skills (Lerman, et al., 2000; Bachmeyer-Lee, 2020), likely placing them in the same category as lecture-based training. In contrast, the modeling component may be crucial in providing staff with a clear example of expected behavior. However, rehearsal and real-time feedback are likely the most critical BST components, as they help shape desired behavior by immediately addressing both correct and incorrect responses (Hoppe et al., 2024; Miltenberger et al., 2017).

To complement the visual analysis in Fig. 7 (Kratochwill et al., 2022), we estimated the effect size of the evocative situations. The results indicate that the intervention produced reliable changes in evocative situations. This is supported by the improvements in children’s response rates during lunchtime (Fig. 8). The figure presents data on listener responses and mand skills, which ideally would have been analyzed separately; however, they were collected as a single data set. The observed patterns align with staff accuracy in implementing evocative situations, demonstrating a clear correlation: when staff apply these strategies correctly, children exhibit more appropriate and expected responses (Greer & McDonough, 1999). Notably, different staff members facilitated lunch sessions and progressively refined their implementation skills throughout the intervention period. These findings highlight the importance of system-wide skill development, ensuring that all unit staff acquire and apply strategies that promote meaningful child participation.

Despite the lack of generalization data for evocative situations, improvements of more than four points were observed in the APERS-P-SE domains of “curriculum and instruction” and “communication” in preschools A and C. These APERS results, along with anecdotal observations, suggest that several staff members in these preschools implemented evocative situations and applied behavior-analytic strategies across multiple situations more frequently after the intervention and at follow-up than before the in-service training.

Anecdotal observations revealed that three children progressed from sitting passively and being fed to requesting food and drinks through picture exchange and vocal mands (i.e., the child in preschool B) and sharing various foods with others seated around the table. Another example of improved quality of life is the child in preschool C, who initially wandered from activity to activity. Over time, the child began requesting different activities as the staff generalized mand training to free-play situations in the classroom, enhancing the overall quality of the teaching and learning environment.

Despite anecdotal observations and changes in children’s responses during lunch, the study found no differences in CARS2 scores after the intervention. Recognizing the limitation of focusing solely on staff training, the project did not include comprehensive assessments of the children’s overall developmental progress. However, this limitation presents an opportunity for future research to incorporate more thorough evaluations using standardized developmental tools, providing a more comprehensive understanding of the intervention’s potential impact.

The participating preschool staff generally provided high ratings on the SVS. However, the results also indicate varying degrees of inclusion following the intervention. Statement five in the SVS (i.e., During the project period, the child has learned new skills and is more included in the children’s group) could have been better operationalized by splitting it into two separate questions. Additionally, there were variations in the outcomes among the different preschools. The staff in preschool C scored higher (mean 6.25) on question five compared to the staff in preschool A (mean 2.5) and B (mean 4.6). A possible explanation may be that children in preschool A and B had CARS2 scores indicating severe symptoms of autism, whereas the child in preschool C had no symptoms of ASD. Additionally, none of the children showed changed CARS2 scores following the intervention. This suggests that the children have pervasive social difficulties, a core symptom of ASD, and that a 17-week intervention may be insufficient to improve inclusion for those with severe autism symptoms on the CARS2.

### Opportunities for Future Research

Implementing evidence-based interventions in high-demand preschool environments presents significant methodological challenges, particularly regarding the complexity of multiple independent variables and intensive coaching protocols. Preliminary findings indicate that unit-wide interventions can be effective when underpinned by a robust pedagogical framework, with the overall quality of general educational practices emerging as a critical factor in intervention sustainability and potential developmental outcomes for children.

The successful implementation of EBPs in preschool settings demands a comprehensive and nuanced approach. Researchers must establish rigorous intake criteria that critically assess leadership support, organizational readiness, and the quality of existing pedagogical practices. This multifaceted evaluation ensures that institutions possess the necessary capacity for sustained intervention and functional change.

A critical line of inquiry focuses on dissecting the components of staff training to understand their relative impact. Researchers need to investigate whether the observed outcomes result from the synergistic combination of in-service training and biweekly coaching or if specific components drive the most significant improvements. This analytical approach necessitates exploring alternative training modalities that balance empirical rigor with practical workplace constraints. Potential alternative training approaches include reduced-frequency coaching models, digital supervision platforms, self-directed theoretical training with targeted supervisor feedback, and automated training programs with adaptive feedback mechanisms. Each of these approaches offers unique advantages in addressing the complex challenges of implementing evidence-based interventions in high-demand educational environments. The ultimate goal remains clear: to develop intervention strategies that not only meet the highest standards of EBP but also provide practical, sustainable solutions that support optimal developmental outcomes for children in preschool settings.

## Conclusion

Staff training and biweekly coaching in three inclusive preschool classrooms increased the accurate implementation of evocative situations during mealtimes and enhanced responding for children. In two preschools, the intervention also improved EIBI quality and the overall teaching and learning environment. The key contribution of the present study lay in its emphasis on training all classroom staff, ensuring they possess sufficient knowledge and skills to provide evocative situations and improve the overall quality of the preschool. This objective was pursued through comprehensive training sessions and biweekly mandatory coaching sessions for all classroom staff. Consequently, this approach facilitated the shaping and development of collaboration between the special educator and classroom staff, particularly in reschool A and C. The positive reception from the staff regarding the collaboration time further underscored the success of these initiatives.

## Data Availability

Owing to privacy considerations for the participants, the data are unavailable beyond what is described and presented in the article.
